# Vitamin D Fortification of Dairy Products in the United Kingdom: What Are the Barriers?^☆^

**DOI:** 10.1016/j.advnut.2026.100645

**Published:** 2026-05-06

**Authors:** Cheuk Lun Wong, D Ian Givens, Daniele Asioli, Anu M Turpeinen, Jia Han, L Kirsty Pourshahidi, Jing Guo

**Affiliations:** 1 School of Food Science and Nutrition, Faculty of Environment, University of Leeds, Leeds, United Kingdom; 2 Institute for Food, Nutrition and Health, University of Reading, Reading, United Kingdom; 3 Department of Agri-Food Economics and Marketing, University of Reading, Reading, United Kingdom; 4 Department of Land, Environment, Agriculture and Forestry, University of Padua, Padua, Italy; 5 Valio Ltd, R&D, Helsinki, Finland; 6 Nutrition Innovation Centre for Food and Health (NICHE), Ulster University, Coleraine, United Kingdom

**Keywords:** cheese, consumer, dairy, fortification, milk, policy, vitamin D, yogurt

## Abstract

Given the inadequate vitamin D intake and status in the United Kingdom population, the Scientific Advisory Committee on Nutrition recognized the difficulty of achieving a reference nutrient intake of vitamin D (10 μg/d) simply from natural food sources. Therefore, additional food vehicles for vitamin D fortification are needed to improve vitamin D intake and status. Dairy products represent a potential vehicle for fortification, given their status as staple foods in the United Kingdom. In recent workshops (October 2024 and March 2025), barriers to vitamin D fortification of dairy products were discussed by a group of experts. The main conclusions from the workshops are presented in this review and position paper and are as follows: *1*) there is convincing evidence of the need to improve vitamin D status. *2*) Furthermore, evidence is required including modeling studies to examine a wide variety of dairy products potentially being fortified using the United Kingdom population data, especially those who are at risk of vitamin D deficiency such as children and young female populations, as well as cost evaluation studies of vitamin D fortification. *3*) More research on consumer preferences toward vitamin D-fortified dairy products is needed. *4*) Vitamin D fortification of dairy products may not be technically difficult, but it may involve additional cost for dairy food manufacturers, such as purchasing specific equipment for vitamin D addition. *5*) The retailing and promotion of vitamin D-fortified dairy products are influenced by various factors such as price and customer perceptions. This review further: *1*) highlights the barriers to the promotion and legislation of mandatory vitamin D fortification policy of dairy products, *2*) reviews current evidence of each barrier, and *3*) identifies research gaps that need to be addressed in the future.

**Statement of Significance**

This position and review paper highlights 5 potential barriers to the legislation and promotion of mandatory vitamin D fortification of dairy products in the United Kingdom, consolidates relevant scientific evidence for policymakers considering such a policy, and identifies research gaps that may need to be addressed before implementation.

## Introduction

It has been known for a long time that vitamin D is an essential micronutrient for skeletal health [1]. Although vitamin D has been associated with benefits for a range of nonmusculoskeletal functions [2,3], including pre-eclampsia [4,5], cancers [6], all-cause mortality [7,8], cardiovascular disease [9,10], immunity [11], and type 2 diabetes [12,13], it is important to note that large randomized controlled trials (RCTs) on vitamin D supplementation in elderly subjects, such as Vitamin D and Omega-3 Trial [14,15], Vitamin D_3_-Omega-3-Home Exercise–Healthy Aging and Longevity Trial [16,17], and Vitamin D and Longevity [18], have reported no benefits on nonmusculoskeletal health outcomes such as all-cause mortality and cancer incidence [19]. Nevertheless, improving vitamin D status remains important primarily to prevent vitamin D deficiency-related skeletal health problems, for example, rickets in children and osteomalacia in adults.

In 2016, the United Kingdom Scientific Advisory Committee on Nutrition (SACN) confirmed that serum 25-hydroxyvitamin D [25(OH)D] concentrations <25 nmol/L were associated with an increased risk of vitamin D deficiency, potentially leading to a higher risk of rickets in young children and osteomalacia in adults [20]. The United Kingdom National Health Service (NHS) defines the vitamin D status of adults into 3 categories of serum 25(OH)D concentration, i.e., <25 nmol/L is deficient and requires treatment, 25 to 50 nmol/L may be inadequate or insufficient in some individuals who will require treatment, and >50 nmol/L is sufficient for most individuals [21]. The SACN [20] also set a reference nutrient intake (RNI) for vitamin D (10 μg/d) for the general population in the United Kingdom aged ≥4 y, including pregnant and lactating females and a safe intake (8.5–10 μg/d) for infants <1 y throughout the year. This amount of vitamin D ensures that 97.5% of the population maintains a serum 25(OH)D concentration ≥25 nmol/L when there is minimal UVB sunshine exposure [20]. However, the vitamin D RNI is relatively conservative compared with the recommended dietary allowance of the Institute of Medicine (IOM; now called the National Academy of Medicine) [22] and the adequate intake of the European Food Safety Authority (EFSA) [23], both recommending 15 μg/d.

The United Kingdom National Diet and Nutrition Survey (NDNS), which assesses the diet, nutrient intake, and nutritional status of the general United Kingdom population according to age group, in 2020 and the latest data in 2025, consistently confirmed the low vitamin D intake and suboptimal vitamin D status in the United Kingdom general population [24,25]. Although the NHS recommends 10 μg/d of vitamin D supplement during winter for individuals aged ≥4 y [21], adherence to supplementation remains low [24,25]. The mean vitamin D intakes from food sources of all age groups ranged from 1.9 to 3.1 μg/d, considerably below the RNI of 10 μg/d, and a substantial percentage of the population had vitamin D deficiency [e.g., serum 25(OH)D concentration <25 nmol/L], especially girls at 11 to 18 y [25]. In addition, evidence has now shown that ≥20 μg/d throughout the year may be needed for the dark-skinned populations to maintain an adequate serum 25(OH)D concentration [26]. Furthermore, there have been reports of a re-emergence of symptomatic vitamin D deficiency and increasing hospitalization rates of rickets in the United Kingdom, particularly among children aged <15 y from ethnic minority groups, including South Asian, Middle Eastern and sub-Saharan [27,28]. These data indicate that actions must be taken to increase vitamin D intake and improve vitamin D status in the United Kingdom population.

The SACN [20] recognized the difficulty of reaching the RNI of vitamin D solely from natural food sources. Therefore, the SACN [29] in 2024 considered vitamin D food fortification as a strategy to increase vitamin D intake and improve vitamin D status in the United Kingdom. After the removal of mandatory vitamin D fortification of margarine in 2013 as part of the initiative of the United Kingdom government to reduce the number of regulations, the vitamin D fortification policy of fat spreads and breakfast cereals is currently voluntary in the United Kingdom [29]. Although there is no vitamin D fortification policy for dairy products in the United Kingdom currently, food manufacturers are permitted to add vitamins and minerals to foods voluntarily as long as they comply with the European Union (EU) Regulation [30]. Vitamin D fortification of dairy products policy has been implemented in a number of countries for a long time, for example, the United States and Finland, and has shown that this strategy is effective in improving population vitamin D intake and status [31,32]. Indeed, recent analysis of newly launched products has reported that dairy products are among the most commonly used food vehicles for vitamin D fortification around the world [33]. Regarding a food vehicle for systematic fortification in the United Kingdom, dairy products may be a suitable option as they have been staple foods in the United Kingdom although there has been a decline in household purchases of dairy products in recent years [34].

Given that there is a lack of a comprehensive overview of the barriers to implementing or promoting voluntary or mandatory vitamin D fortification of dairy products in the United Kingdom, this present review and position paper aims to: *1*) highlight the potential barriers which may hinder the promotion and legislation of mandatory vitamin D fortification of dairy products in the United Kingdom, *2*) review evidence of the highlighted barriers, and *3*) provide information on the availability and vitamin D concentrations of vitamin D-fortified dairy products in United Kingdom supermarkets. This paper is important for advancing the subject of food fortification and public health nutrition as well as identifying research gaps and providing future directions to overcome the barriers.

## Methods

### Closed consensus workshops

This review and position paper is based on presentations, discussions, and conclusions from workshops on “overcoming the barriers to maximize the commercial potential of vitamin D-fortified dairy products in the United Kingdom.” During the 2 workshops (October 2024 and March 2025), topics including dairy product fortification, vitamin D and health, consumer preferences, legislation, and retailing of vitamin D-fortified dairy products were presented by experts (JG, DIG, AMT, DA, LKP, representative from Arla Foods United Kingdom, and representative from Marks & Spencer United Kingdom). Each presentation was deeply discussed and challenged.

### Commercial vitamin D-fortified dairy products online search

To supplement the workshop outcomes, this search aimed to examine 3 aspects of vitamin D-fortified dairy products, including but not limited to milk, yogurt, and cheese, in the United Kingdom: their market availability, vitamin D concentrations, and their potential contribution to the vitamin D RNI. The search for commercial vitamin D-fortified dairy products was conducted in October 2024. The search was conducted on the websites of 10 main United Kingdom supermarkets, covering ∼95% of the grocery market share in 2024 [35], and 1 family-run dairy foods business. To confirm whether a product was fortified with vitamin D, its ingredient list was examined to check if vitamin D was listed on the ingredients [36]. The percentage contribution to the vitamin D RNI (10 μg/d) of the vitamin D-fortified dairy products was calculated based on the portion size suggested by the British Nutrition Foundation (BNF) [37], which is complementary to the Eatwell Guide guidance, including 200 mL of whole/semi-skimmed/skimmed milk and 120 g of plain/low-fat yogurt. For simplicity, portion sizes of 200 mL/d for milk and 120 g/d for yogurt, yogurt drinks, fromage frais, and dairy desserts were used in the calculations.

**TABLE 1:** Vitamin D concentrations of fortified fermented dairy products and desserts searched online from supermarkets and family-run business in the United Kingdom^1^

Retailer (*n*)	Brand^2^ dairy type	Vitamin D (μg/100 g)	% of vitamin D RNI (10 μg/d)^3^
	Brand A	1.91	22.9
1	Yogurt	0.50	6.0
4	Yogurt drink	2.09	25.0
	Brand B	0.70	8.3
1	Custard	0.68	8.2
	Yogurt drink	0.71	8.5
	Brand C	0.75	9.0
1	Yogurt drink	0.75	9.0
	Brand D	0.75	9.0
1	Kefir	0.75	9.0
	Brand E	3.40	40.8
2	Yogurt	3.40	40.8
	Brand F	1.30	15.6
2	Yogurt	1.30	15.6
	Brand G	1.77	21.3
1	Yogurt	1.77	21.3
	Brand H	1.93	23.1
5	Cheesecake	1.79	21.5
1	Yogurt	1.93	23.2
	Brand I	0.88	10.6
3	Fromage frais	0.90	10.8
2	Yogurt drink	0.83	10.0
	Brand J	2.90	34.8
1	Fromage frais	2.90	34.8
	Brand K	2.90	34.8
2	Fromage frais	2.90	34.8
	Brand L	2.02	24.2
4	Fromage frais	2.20	26.4
1	Yogurt	2.90	34.8
2	Yogurt drink	0.75	9.0
	Brand M	2.15	25.8
2	Yogurt	2.15	25.8
	Brand N	0.75	9.0
1	Yogurt drink	0.75	9.0
	Brand O	0.91	10.9
4	Yogurt	0.91	10.9
	Brand P	3.21	38.6
3	Fromage frais	2.90	34.8
	Yogurt drink	3.40	40.8
Overall vitamin D			
Mean (μg/100 g)		1.77	21.3
Range (μg/100 g)		0.50–3.40	—

Abbreviations: BNF, British Nutrition Foundation; RNI, reference nutrient intake; Vit, vitamin.

1 Data presented in mean value.

2 Each letter represents a different brand.

3 Calculation based on the portion size (120 g/d of yogurt) suggested by the BNF [37].

**TABLE 2:** Vitamin D concentrations of fortified liquid milk searched online from supermarkets and family-run businesses in the United Kingdom^1^

Retailer (*n*)	Brand^2^ Dairy type	Vitamin D (μg/100 mL)	% of vitamin D RNI (10 μg/d)^3^
	Brand Q	2.20	44
4	Whole milk	2.20	44
	Brand R	2.20	44
1	Semiskimmed Milk	2.20	44
	Whole milk	2.20	44
	Brand S	0.26	5.2
1	Whole milk	0.26	5.2
Overall vitamin D			
Mean (μg/100 mL)		1.92	38.5
Range (μg/100 mL)		0.26–2.20	—

Abbreviations: BNF, British Nutrition Foundation; RNI, reference nutrient intake; Vit, vitamin.

1 Data presented in mean value.

2 Each letter represents a different brand.

3 Calculation based on the portion size (200 mL/d of milk) suggested by the BNF [37].

### The necessity to improve vitamin D status in the United Kingdom

#### Vitamin D intake and status of the United Kingdom population

It is believed that suboptimal vitamin D status is extensive in adults and children in many parts of the world, and Cashman et al. [38] confirmed that within Europe, the prevalence of vitamin D deficiency represents a major health risk requiring a major public health initiative to resolve it, particularly to prevent skeletal health problems such as rickets and osteomalacia.

The latest data on vitamin D intake and status in the United Kingdom population come from the NDNS reports (years 9–15) [24,25] and are summarized in Table 3. Vitamin D intake is consistently and substantially lower than the RNI, with females tending to have lower mean intakes than males. In relation to the English Indices of Multiple Deprivation (EIMD), which measure relative levels of deprivation in small areas or neighborhoods in England, the 2025 NDNS [25] data also suggested that vitamin D intake (μg/d) may be influenced by the level of deprivation, especially among those aged ≥65 and 11 to 18 y. In general, individuals from the less deprived group had higher mean daily consumption of vitamin D in their respective age groups. For those aged ≥65 y, the least deprived consumed a mean of 3.1 μg/d of vitamin D, whereas the most deprived had a mean vitamin D intake of 2.3 μg/d. Similarly, among those aged 11 to 18 y, the least deprived had a mean consumption of 2.3 μg/d, whereas the most deprived had 1.9 μg/d. Regardless of the scenario, the daily vitamin D intake is far from optimal (RNI of 10 μg/d). A substantial proportion of all ages other than children have serum 25(OH)D <25 nmol/L, and the proportion will increase in the winter. It is worth noting that the percentage of adolescent girls (11–18 y) with serum 25(OH)D concentration below 25 nmol/L increased from 17% to 26%, while that of children (4–10 y) rose from 2% to 10% (Table 3), indicating children and young female populations may be the most vulnerable groups for vitamin D deficiency. Moreover, given that an adequate vitamin D status [i.e., serum 25(OH)D concentration >50 nmol/L] is a target of the United Kingdom NHS and EFSA for most individuals [21,23], meaning that the percentage of the population <50 nmol/L would be substantially higher than those <25 nmol/L (Table 3). Overall, these data strongly suggest that the vitamin D status and intake of the United Kingdom population are at best marginal.

**TABLE 3:** Vitamin D intake and status in the United Kingdom from NDNS (years 9–11 and 12–15) [24,25]

Population group (y)	Mean vitamin D intake (μg/d)			% with serum 25(OH)D <25 nmol/L^3^		
	Years 9–11^1^	Years 12–15^2^	Difference	Years 9–11	Years 12–15	Difference
Children 1.5–3	2.4	1.9	–0.5	ND	ND	ND
Children 4–10	2.3	2.1	–0.2	2	10	+8
Boys 11–18	2.4	2.4	0	21	20	–1
Girls 11–18	2.1	2.0	–0.1	17	26	+9
Males 19–64	3.2	2.9	–0.3	18	20	+2
Females 19–64	2.6	2.3	–0.3	15	17	+2
Males 65+	3.6	3.1	–0.5	13	17	+4
Females 65+	2.8	2.7	–0.1	13	8	–5
Males 75+	4.0	2.7	–1.3	ND	ND	ND
Females 75+	2.8	2.9	–0.1	ND	ND	ND

Abbreviations: 25(OH)D, 25-hydroxyvitamin D; ND, not determined; NDNS, National Diet and Nutrition Survey.

1 Excludes supplements, values with supplements were 2.9–10.1 μg/d.

2 Excludes supplements, values with supplements were 3.4–11.3 μg/d.

3 Values in January–March were 7%, 36%, and 31% of children aged 4–10, children aged 11–18 y, and adults aged 19–64 y, respectively (years 9–15 combined).

Recently, the SACN [29] produced a report on “Fortifying foods and drinks with vitamin D.” One of the aims was to incorporate new evidence on vitamin D status and related factors since its 2016 review. A key conclusion was that the earlier advice to take a daily supplement of 10 μg, particularly during autumn and winter months, has had limited impact since a substantial percentage of the population still had low vitamin D status [serum 25(OH)D <25 nmol/L]. The SACN [29] concluded that “this suggests that other strategies may be necessary to achieve recommended intakes of vitamin D.” One option considered was a food fortification strategy but recommended further consideration and a modeling exercise “to identify suitable fortification vehicles that would reach all population groups in the UK and assess safe levels of fortification.”

#### Dietary sources of vitamin D

The RNI of vitamin D clearly relates to all dietary sources including natural foods, fortified foods, and supplements. There are a few foods that are naturally rich in vitamin D_3_ and those that contain valuable amounts are exclusively of animal origin (e.g., meat, oily fish, and egg yolk). Besides, there are no known plant sources of vitamin D, although some mushrooms (a type of fungi) grown under specific conditions are a source of vitamin D_2_ although this is generally less potent than vitamin D_3_ [29,39]. In the United Kingdom, the top 4 dietary sources of vitamin D for adults (19–64 y) are meat/meat products, eggs/egg dishes, fortified cereals, and fish/fish dishes, which provide 25%, 16%, 11%, and 10% of dietary vitamin D, respectively [25]. Habitual dietary patterns will therefore have a substantial impact on vitamin D intake.

A systematic review of 15 studies involving adults examined vitamin D intake according to diet type [40]. The reported mean intakes were 5.25, 4.17, 2.67 and 1.52 μg/d for pesco-vegetarians, meat eaters, vegetarians and vegans, respectively. In addition, Hedegaard et al. [41] reported vitamin D intakes of 3.3, 4.2, 1.7, and 1.1 μg/d by nonsupplemented pregnant Danish females following omnivorous, vegetarian (fish/poultry), vegetarian (lacto/ovo) and vegan dietary patterns, respectively. All of the above vitamin D intakes were substantially lower than the recommended 10 μg/d, predictably with the lowest intakes in vegetarian and vegan diets although Hedegaard et al. [41] showed that individuals taking vitamin D supplements had mean intakes above 10 μg/d, except the vegans whose mean intake was only 6.2 μg/d.

In terms of vitamin D status based on serum 25(OH)D concentrations, Neufingerl and Eilander [40] reported data from 11 studies showing that status was higher in pesco-vegetarians (72.3 nmol/L), meat eaters (65.5 nmol/L), and semivegetarians (64.5 nmol/L), than vegetarians (57.0 nmol/L) and vegans (54.8 nmol/L). However, it is worth noting that there was only 1 study each for pesco- and semivegetarians. Besides, 4 studies reported in [40] found that vitamin D deficiency (<25 nmol/L) was low in meat-eaters and pesco-vegetarians (0%–6%) but higher in vegetarians (0%–33%) and especially vegans (3%–67%). Overall, it was concluded that the highest rate of vitamin D deficiency was in vegetarians and vegans. This finding was supported by a study of Finnish females [42], which also reported significantly lower bone mineral density in the lumbar spine region and femoral neck in vegans compared with omnivores.

#### The decline in meat consumption: the largest source of dietary vitamin D in the United Kingdom

Given the evidence that meat is the greatest dietary source of vitamin D in the United Kingdom, it is important to understand the concentrations of vitamin D in the different types of meat and trends in meat consumption. Schmid and Walther [43] reviewed the food composition databases of Denmark, France, Germany, Switzerland, Canada, and the United States which combined gave the following ranges of vitamin D concentrations (μg/100 g): 0.0 to 0.9 for beef, 0.1 to 2.3 for pork, 0.1 to 6.1 for lamb, and 0.0 to 1.4 for poultry. These values are in broad agreement with those in the United Kingdom Food database although its maximum value for lamb is 0.8 μg/100 g and chicken and turkey 0.5 μg/100 g [44]. Considerably higher concentrations are seen in offal including liver, kidneys, fat and poultry skin. In a study with 4 cuts of beef and lamb, positive relationships were seen between fat percentage and vitamin D_3_ concentration before and after cooking, but not for 25(OH)D_3_ [45]. Schmid and Walther [43] also reported that vitamin D concentration is not much affected by processing and cooking since vitamin D is rather heat and oxygen-tolerant.

**FIGURE 1:**
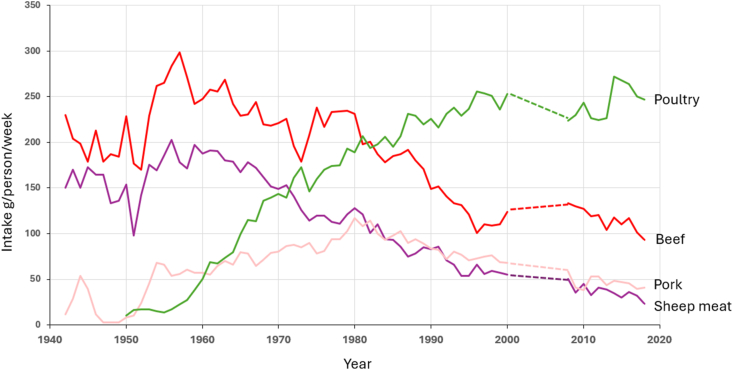
Trends in consumption of meat in the United Kingdom from 1940 to 2020. The trends from 1940 to 2000 are based on data from the United Kingdom National Food Survey. From 2000 to 2019, trends are derived from Stewart et al. [46] which used data from NDNS. The gap between the 2 data sources is denoted by the dotted line. NDNS, National Diet and Nutrition Survey.

As seen in Figure 1, United Kingdom consumption of beef and sheep meat/lamb has declined overall since about 1955 and has continued to decline in recent years. Pork consumption increased from about 1950 to 1980 but has since declined, although it has remained stable over the last 10 y. Consumption of other red meat and offal is low compared with other meat categories, but has also tended to decline in the last 10 y. These general reductions in red meat consumption will have lowered their contribution of vitamin D, and this is likely to reduce further in the coming years. Poultry meat consumption has increased substantially since the late 1950s but has remained relatively stable over the last 10 y with only a small increasing trend (P-trend = 0.0024) [46].

Overall, given the relative stability of poultry meat and pork consumption and the continued decline in beef and lamb consumption, the net effect will be a continued reduction in the dietary intake of vitamin D from meat. Given the already low intakes of iron by United Kingdom adolescent girls [47] linked with a chronic decline in red meat consumption, it is likely that this will be the most vulnerable group to a further decline in vitamin D intake. It is important to realize that this group also has substantially suboptimal intakes of calcium and magnesium already making them at risk of suboptimal bone development in the adolescent period, which will be exacerbated by further reductions in vitamin D.

#### Dietary protein transition: from animal to plant sources

It is also noteworthy that there are increasing concerns about the consumption of animal-derived foods because of their believed negative effects on animal welfare, the environment and human health. This has given rise to an increased interest in replacing at least a proportion of dietary protein of animal origin with that from plants. Given what is known about the dietary sources of vitamin D, this so-called dietary transition will also lead to lower vitamin D dietary intakes (see review published by Givens [47]). There is already evidence that dietary transition to vegetarian and vegan dietary patterns leads to reduced vitamin D intake. Some of the evidence of low vitamin D intake and status linked to vegetarian and vegan dietary patterns was noted earlier [[40], [41], [42]] and these findings are supported by the prospective European Prospective Investigation into Cancer and Nutrition (EPIC)-Oxford study of Tong et al. [48] which found that after adjustment for socioeconomic factors, lifestyle confounders, and BMI, vegans had significantly increased risks for total fractures [hazard ratio (HR) = 1.43, 95% confidence interval (CI): 1.20, 1.70], hip (HR = 2.31, 95% CI: 1.66, 3.22) and leg fractures (HR = 2.05, 95% CI: 1.23, 3.41) relative to meat eaters. Vegetarians also had an increased risk of hip fractures (HR = 1.25, 95% CI: 1.04, 1.50).

**FIGURE 2:**
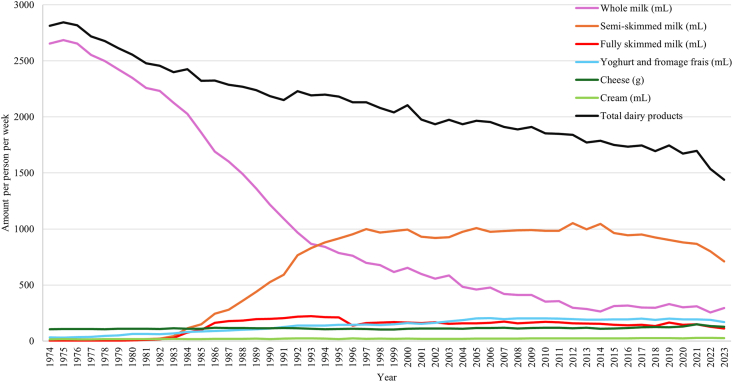
Trends in the quantity of dairy products purchased by United Kingdom households from 1974 to 2022. The trends are based on data from the United Kingdom Family Food Survey 2023 [34].

**FIGURE 3:**
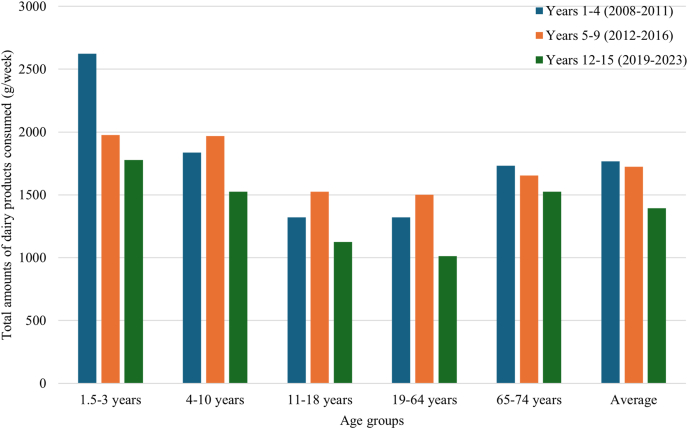
Total amounts of dairy products consumed (g/wk) in the United Kingdom using data from NDNS years 1–4, 5–9, and 12–15. Dairy products are defined as whole milk (3.8% fat), semiskimmed milk (1.8% fat), 1% fat milk, skimmed milk (0.5% fat), other milk and cream, cheese, yogurt, fromage frais, and other dairy desserts. NDNS, National Diet and Nutrition Survey.

In relation to dairy products, Figures 2 and 3 illustrate trends in the quantity of dairy products purchased by United Kingdom households and the total amount consumed in the United Kingdom, respectively. Although household purchases of dairy products have been declining since the 1970s, particularly whole milk, dairy products remain widely consumed across the United Kingdom population. This suggests that dairy products, particularly semiskimmed milk, may still represent effective vehicles for vitamin D fortification. Nevertheless, the long-term downward trend in household purchases of dairy products (Figure 2) may limit the overall effectiveness of vitamin D fortification of dairy products and its ability to reach the wider population. This decline may partly reflect the ongoing dietary transition from animal-derived proteins to plant-based alternatives [47], meaning that individuals following vegetarian or vegan diets may not benefit from vitamin D-fortified dairy products [49]. Although the latest NDNS in 2025 [25] reported that ∼3% of the United Kingdom population follow a vegetarian diet, and 0.4% follow a vegan diet, the actual percentage of nonconsumers of dairy products may be underestimated given that some omnivores would avoid dairy products due to lactose intolerance or personal preferences. Given that achieving broader population coverage is a key goal highlighted by the SACN [29], complementary fortification strategies involving additional food vehicles (e.g., plant-based milk products) should also be considered [49], particularly among nonconsumers of dairy products.

### Barrier 1: uncertainty in the policy process in relation to mandatory vitamin D fortification

#### Insights from mandatory folic acid fortification in the United Kingdom

Given the limited research on the challenges in food fortification from a policy-making perspective, the case of mandatory folic acid fortification in the United Kingdom serves as a useful example for identifying potential obstacles which may arise when promoting mandatory vitamin D fortification of dairy products.

Several concerns can be highlighted in relation to vitamin D fortification. First, the legislation process is likely to be lengthy. For example, it took ∼5 y from the public consultation (2019) [50] to the official announcement of mandatory folic acid fortification (2024) [51]. More importantly, the United Kingdom government must first recognize and acknowledge the need for such action before initiating the legislation process. The journey of the SACN’s recommendation for folic acid fortification in 2006 and 2009 [52] to the official announcement of the legislation in 2024 [51] spanned almost 20 y despite strong evidence demonstrating its efficacy in preventing neural tube defects (NTDs) [53]. The prolonged legislation process creates a gap during which at-risk groups as well as the wider United Kingdom population with suboptimal vitamin D intake and status remain vulnerable. Second, uncertainty remains regarding which dairy products should be fortified with vitamin D. A modeling study would provide valuable evidence to evaluate the effectiveness of fortifying different dairy products with vitamin D. It is also known that countries with a vitamin D fortification policy covering a wide variety of dairy products contribute significantly more to vitamin D intake than countries with a partial coverage and without a fortification policy [54]. For folic acid fortification, despite criticisms that the current plan may be ineffective [55], only nonwholemeal wheat flour is fortified rather than all types of flour in the United Kingdom [51], which may be less beneficial to nonconsumers of food products that are made from nonwholemeal wheat flour. Moreover, folic acid fortification will not begin until the end of 2026 [51]. This example shows how difficult it will be to get the government in the right direction and speed in terms of the fortification approach. Third, what evidence is needed for the government to be convinced that vitamin D fortification is essential? The reason that held the folic acid fortification back was that the government prioritized unproven risks over proven benefits [56]. As a result, only nonwholemeal wheat flour is to be mandatorily fortified with a relatively low concentration of folic acid (0.25 mg/100 g) [55,57], and may only reduce 20% of NTD cases annually in the United Kingdom [58].

#### Voluntary compared with mandatory fortification: a United Kingdom-wide or unilateral approach?

There are uncertainties regarding whether vitamin D fortification of dairy products should be voluntary or mandatory, and whether it should be implemented United Kingdom-wide or on a unilateral basis. Although mandatory fortification may be more effective compared with voluntary fortification as a public health intervention [59,60], more concerns about mandatory vitamin D fortification were raised by the public such as consumer choice and toxicity [61]. The decision between voluntary and mandatory fortification must be based on various factors, particularly the prevalence of nutrient deficiency and the risk of excessive intake among the population [62]. Furthermore, given the devolution in the United Kingdom, health policies are determined independently by governing bodies in Scotland, Wales, Northern Ireland, and England [63]. This is particularly relevant given the historical reluctance to establish mandatory folic acid fortification at a national level. A notable example is that Food Standards Scotland considered proceeding with mandatory folic acid fortification of flour in Scotland unilaterally because of the lack of progress in the United Kingdom in 2016 [52].

### Baqs,'p[2r3rrier 2: consumer preferences for vitamin D fortification and vitamin D-fortified dairy products

It is important to identify factors that influence consumer preferences for vitamin D-fortified dairy products as getting a better understanding of those factors may help to identify and overcome barriers that hinder consumers from buying these products. A few studies have examined the consumer preferences and willingness to pay (WTP) toward vitamin D-fortified foods in the United Kingdom [61,[64], [65], [66], [67], [68]].

Clark et al. [64] conducted a survey and found that most participants had low awareness of the risk factors associated with vitamin D deficiency. Despite the results showing that 55% of the participants believed it was a good idea to fortify foods with vitamin D, 43% and 10% were unsure and disagreed with this idea, respectively. This could be partially explained by the fact that 73% of the participants would like to obtain vitamin D from natural sources. They also found that participants who were aware of the importance of obtaining vitamin D through diet and/or aware of the health benefits of achieving optimal vitamin D levels had higher intentions to buy vitamin D-fortified foods.

O’Connor et al. [65] conducted a cross-sectional study with the United Kingdom adults regarding the knowledge, attitudes, and perceptions toward vitamin D. They reported some consumer concerns about fortified foods, including “fear of vitamin overdose,” “lack of choice,” “a desire to avoid processed food products,” “a belief that relying on fortified foods may eventually lead to less nutrition understanding,” and “a desire to achieve vitamin D status through natural sources.” Another online survey was conducted by Erhard et al. [69] on adults in Denmark, Iceland, and the United Kingdom regarding the purchase intention of vitamin D-fortified drinks. They identified 3 segments, including low, medium, and high purchase intention of vitamin D-fortified drinks. The results showed that the differences in purchase intention of vitamin D-fortified drinks may be due to age and dietary habits, and varied among European countries. Erhard et al. [69] suggested that older respondents may have considered vitamin D-fortified drinks novel and therefore had a lower purchase intention. This could be partially explained by the fact that older consumers may accept familiar foods more easily and are less open to novel foods [70,71]. Additionally, the results also showed that respondents, who had a predominantly plant-based diet, may be more open to vitamin D-fortified drinks, it may be because they had a higher awareness of nutritional deficiency risk. The results implied that more effort may be needed to increase the purchase intention of omnivores for vitamin D-fortified foods [69].

Trade-offs between consumer preferences and the benefits of fortified foods can be observed. Areal and Asioli [68] investigated the consumer preferences and WTP for vitamin D-fortified eggs. They found that factors including the production method and price of the fortified eggs, and the age may influence their preferences and WTP. Results also showed that those who preferred organic eggs or preferred environmental and ethical aspects were less likely to choose eggs fortified with vitamin D. Moreover, consumers had a higher WTP for organic eggs than eggs labeled with the claim “vitamin D added.” This demonstrated a tradeoff between organic, ethical considerations and health benefits. Furthermore, in a focus group study by Embling et al. [67], the taste and texture of the fortified foods also seemed to be a tradeoff for the additional health benefits.

Price is also a barrier for consumers to buy fortified foods [67,72] as discovered in a German study [72]. The most common reason for opposing vitamin D-fortified products was “too expensive.” Thus, further research on the consumer WTP for vitamin D-fortified dairy products in the United Kingdom population is needed.

Regarding the attitude and acceptance toward vitamin D fortification, Clark et al. [61] conducted 2 United Kingdom–based pilot studies in 5 at-risk vitamin D deficiency groups. Results showed that there was a lack of knowledge and misconceptions of at-risk factors for vitamin D deficiency, including sunlight exposure, dietary intake, darker skin pigmentation, sources of vitamin D and the health benefits associated with optimal consumption. Moreover, a lower awareness of vitamin D-fortified foods was found in the at-risk groups (60%) compared with the non-at-risk group (71%). Besides, younger participants had a positive attitude toward vitamin D fortification and found that participants had more positive opinions of voluntary fortification than mandatory fortification due to the concerns of consumer choice and the toxicity of vitamin D.

An association between general attitudes toward vitamin D fortification and intention to purchase vitamin D-fortified food was examined in Danish adults [66]. The results showed that participants considered dairy products including milk, yogurt, and cheese to be appropriate for food fortification, which may be due to the previous implementation (in 2011) of vitamin D fortification of certain dairy products. However, it was withdrawn in 2014 due to poor sales [66]. In comparison to the United Kingdom where breakfast cereals were the most preferred when it comes to vitamin D fortification [61], this may be related to the fact that voluntary vitamin D fortification of breakfast cereals has been implemented since 2006 [73]. Consumer purchase intention for vitamin D-fortified food was positively associated with the perceived benefits and influenced by the level of problem awareness of vitamin D deficiency and the appropriateness of fortified foods.

There were concerns of sensory properties raised by consumers such as taste and texture [74], which could be a barrier for consumers to accept vitamin D-fortified foods. Cashman [74] reviewed studies evaluating the sensory properties of vitamin D-fortified yogurts [75,76], cheese [[77], [78], [79]], and milk [75], and concluded that vitamin D fortification is unlikely to affect the sensory properties of foods. In addition, a more recent sensory study that investigated the sensory properties of vitamin D-fortified yogurt [80] was similar with previous findings which showed that vitamin D fortification had no adverse effects on sensory attributes (i.e., taste, texture, aroma, appearance, and overall acceptance). Thus, given that vitamin D fortification is unlikely to alter the sensory properties of milk, yogurt, and cheese regardless of the fortification level, this evidence may be useful for education and promotion so that consumer concerns regarding the sensory properties of fortified dairy products can be addressed.

Despite there being few studies conducted in the United Kingdom about consumer preferences for vitamin D-fortified foods and vitamin D fortification, a small sample size of the United Kingdom population included was acknowledged [61,64,65]. Therefore, further United Kingdom–based studies are needed to confirm the findings using a larger sample size and representative data from the United Kingdom population, and further studies of consumer preferences and attitudes toward vitamin D-fortified dairy products are also needed.

### Barrier 3: unclear impacts on milk processors and current raw milk processing plants

Direct vitamin D fortification is likely to be implemented given there are existing examples such as Finland, United States, and Canada where vitamin D is fortified to several common dairy foods, including yogurts, cheese, and milk [54,81,82]. Although the vitamin D content of milk could potentially be increased through biofortification [83], its effectiveness at the population level remains uncertain. Therefore, direct fortification is likely to be the primary approach. The raw milk processing procedures can be generally summarized in 5 key steps including *1*) separation, *2*) standardization, *3*) homogenization, *4*) pasteurization or ultraheat treatment (UHT), and *5*) cooling [84,85]. To produce yogurt and cheese, additional steps are needed after heat treatment of raw milk, for example, fermentation [85,86]. Besides, the milk used for cheese production is separated from the milk intended for liquid sale [84]. For direct fortification of milk, vitamin D premix for fortification is generally available in 2 forms, including oil-based and water-dispersible formulations. Oil-based premix must be added to the flow of milk after cream separation, whereas water-dispersible premix can be incorporated at any stage in the milk flow, but the fortification usually occurs after separation and standardization of fat and before heat treatment (i.e., pasteurization or UHT) as this allows the vitamin D to distribute evenly during homogenization [87]. In addition, appropriate equipment is needed for the addition of vitamin D such as metering pumps [87], which represents a significant capital investment. The vitamin D premix needs to be stored under specific conditions to maintain its potency, creating additional challenges for manufacturers [87]. Small-scale dairy producers may face particular difficulties with both storage costs and limited production space. In addition, the cost of sourcing the vitamin D premix from commercial suppliers may also be a consideration. Overall, although fortifying dairy products with vitamin D during processing may not be complex, the storage requirement for vitamin D premix and the necessary equipment for fortification could add to the manufacturing costs.

### Barrier 4: challenges in retailing and promoting vitamin D-fortified dairy

#### The availability of vitamin D-fortified dairy products in the United Kingdom

As shown in Table 1, vitamin D-fortified fermented dairy products and desserts from 16 brands are currently (October 2024) available across 7 United Kingdom supermarkets. These products included yogurt (*n =* 8), yogurt drink (*n =* 7), custard (*n =* 1), cheesecake (*n =* 1), fromage frais (*n =* 5), and kefir (*n =* 1). These products contained a mean vitamin D concentration of 1.77 μg/100 g (range: 0.50–3.40 μg/100 g), contributing a mean of 21.3% to the daily vitamin D RNI. Furthermore, Table 2 shows that vitamin D-fortified liquid milk was available in 5 retailers, with only 3 brands providing vitamin D-fortified milk. There were 2 types of fortified milk including whole (*n =* 3) and semiskimmed milk (*n =* 1). The mean concentration of vitamin D in these products was 1.92 μg/100 mL (range: 0.26–2.20 μg/100 mL). The mean RNI contribution of these products was 38.5%. Although both fermented dairy products and milk have similar mean vitamin D concentrations, 1.77 μg/100 g and 1.92 μg/100 mL, respectively, consuming vitamin D-fortified milk contributed more to the RNI. The difference reflects the larger portion size for milk (200 mL/d) compared with yogurt (120 g/d). Although the RNI percentage contribution provides insight into the proportion of daily vitamin D intake based on theoretical assumptions, the effect on serum 25(OH)D concentration cannot be predicted as the dose–response between vitamin D intake and serum 25(OH)D is nonlinear [88]. Therefore, to estimate the amount of vitamin D needed to be fortified in foods and the effect on serum 25(OH)D concentration, modeling exercises would be needed.

More vitamin D-fortified fermented dairy products than vitamin D-fortified milk were available in the supermarkets (TABLE 1, TABLE 2) where most products were found available in an online-based supermarket. It is worth mentioning that Asda launched vitamin D-fortified milk in 2017 and 2018 [89,90]. However, information about the products could not be found on its website. On the other hand, no vitamin D-fortified dairy products were identified in low-cost supermarkets. Given that socioeconomic status is associated with consumers’ choice of retailer, Pechey and Monsivais [91] found that 31% of low-cost supermarket users were from the lowest occupational social class group and 15% were from the highest group. Thus, this may be a potential socioeconomic inequality in accessing vitamin D-fortified dairy products if the fortification policy is voluntary. Although mandatory vitamin D fortification of dairy products might prevent such inequality, it would also eliminate consumers’ choice to opt for unfortified dairy products.

#### Attitudes of retailers toward vitamin D fortification of dairy products

To our knowledge, no study has investigated retailers’ attitudes toward food fortification which may be a potential research gap. However, limited evidence exists linking retailers’ perspectives to healthy food retail. Martinez et al. [92] examined supermarket retailers’ perspectives on healthy food retail strategies in the United States to understand the decision-making process of supermarket retailers and barriers to promoting more healthy products. As a result, 20 retailers were included in the analysis after contacting 112 supermarkets in New York State (response rate of 18%). Results showed that perceived customer demand and the product availability of suppliers and deals were key factors in influencing in-store practices such as product selection, placement, pricing, and promotion. Regarding product selection, supplier suggestions and incentives, for example, discounts on bulk orders, were factors that influenced the decisions of retailers [92]. Martinez et al. [92] demonstrated the complex relationship between consumer, supplier, and retailer that contributes to the in-store activity (i.e., selection and promotion of healthier food) and probably affects the food choice of consumers. In our case, further research is needed to identify the drivers and barriers influencing United Kingdom retailers (e.g., large-chained supermarkets) in the selection and promotion of vitamin D-fortified dairy products.

Despite limited evidence, healthy food policies or campaigns launched by retailers may reflect their attitudes toward vitamin D-fortified foods or vitamin D fortification. For example, Asda launched a vitamin D-fortified semiskimmed milk in 2017 [89], and a new multivitamin milk was also launched in 2018 [90]. However, both products are no longer available on its website. Apart from Asda, other United Kingdom large-chain supermarkets have shown commitments to improve the health of the customers, such as providing healthier foods, as part of their company policy [[93], [94], [95], [96], [97]]. Although large-chain retailers may show a positive attitude toward mandatory or voluntary vitamin D fortification of dairy products, perceived customer demand, the product availability of suppliers and deals may limit the availability of vitamin D-fortified dairy products in stores [98]. For example, this happened in Denmark where vitamin D-fortified dairy products were removed due to poor sales in 2014 [66]. It is also worth noting that vitamin D-fortified dairy products have been available in the United Kingdom supermarkets (TABLE 1, TABLE 2) given there is no vitamin D fortification policy for dairy products in the United Kingdom, but food manufacturers are permitted to voluntarily add vitamins and minerals to foods unless it is restricted such as unprocessed foodstuffs in compliance on EU Regulation No.1925/2006 [30,73]. Moreover, a significantly smaller number of vitamin D-fortified milk products is available in the supermarkets compared with that of vitamin D-fortified yogurts (Table 2), which may imply that the demand for fortified milk is low. It is suggested that economic incentives may be important to encourage consumers to buy healthy products and should be led by the government [98] and as discussed in barrier 2, educational campaigns would also be helpful to increase consumers’ acceptance of vitamin D-fortified dairy products by addressing their concerns, which may potentially increase the demand for vitamin D-fortified dairy products.

### Barrier 5: gaps in evidence needed for policymakers

This section reviews evidence that may be required for policymakers based on the example of mandatory folic acid fortification in the United Kingdom, including: *1*) vitamin D safety and toxicity; *2*) the necessity of systematic vitamin D fortification; *3*) the efficacy of vitamin D fortification of dairy products on improving vitamin D status; *4*) real-world example of vitamin D fortification of dairy products; *5*) modeling study of vitamin D fortification of dairy products using representative United Kingdom population data; *6*) cost-effectiveness evaluation of vitamin D fortification of dairy products; and *7*) consumer preferences toward vitamin D-fortified dairy products (already discussed in barrier 2). As discussed earlier, there is consistent evidence that vitamin D intake and status remain suboptimal in several United Kingdom population groups, suggesting the need for vitamin D fortification in the United Kingdom. This evidence may be important for informing the consideration of implementing effective public health interventions to improve the population vitamin D status and reduce the risk of vitamin D deficiency.

#### Vitamin D safety and toxicity

The evidence of the safety of vitamin D, especially at high intakes, is crucial as this may contribute to the decision on the concentration for food fortification. Galior et al. [99] reviewed rare cases of vitamin D intoxication or hypervitaminosis D due to inappropriate administration, incorrect prescription, or manufacturing errors. The reported serum 25(OH)D concentrations in these cases ranged from 375 to 3050 nmol/L, serum calcium concentrations were between 11.1 and 23.1 mg/dL, and the doses of vitamin D consumed ranged from 1250 to 65,100 μg/d, which are hundreds of times more than the vitamin D RNI of 10 μg/d in the United Kingdom [20]. Therefore, it is unlikely to consume such a high dose of vitamin D that could potentially cause vitamin D intoxication or hypervitaminosis D simply from natural food sources or fortified foods. Although the mechanisms of hypervitaminosis D are not fully understood, 1 proposed explanation is that excessive vitamin D intake markedly elevates serum 25(OH)D concentration that exceeds the vitamin D binding protein (DBP) binding capacity. Once DBP becomes saturated, unbound vitamin D metabolites may enter target cells and directly impact gene transcription [100], contributing to the adverse physiological effects associated with toxicity.

Regarding the safety limit of vitamin D intake, Table 4 summarizes the recommended intake and upper limits (ULs) of vitamin D established by the SACN [20], IOM [22], and EFSA [[101], [102], [103]]. In general, vitamin D intakes that remain within the ULs are considered safe (Table 4). The SACN did not establish ULs for vitamin D intake [20], but considers the ULs set by the EFSA in 2012 appropriate [101]. In 2018, the EFSA updated the UL of vitamin D for infants, in which the UL of 25 μg/d for ages 0 to 6 mo remained unchanged, but increased the UL for ages 7 to 11 mo from 25 to 35 μg/d [102]. In addition, a no-observed-adverse-effect-level for vitamin D was previously set at 250 μg/d in 2012 [101]. However, the EFSA reassessed the existing literature on the effect of vitamin D on hypercalcemia in 2023 and decided that a lowest-observed-adverse-effect-level for vitamin D should be set at 250 μg/d based on 2 human RCTs [103]. The recommended intake of vitamin D (10 μg/d) set by the SACN is lower than that set by the IOM and EFSA (both 15 μg/d). Similarly, the ULs for children aged 6 to 12 mo (37.5 μg/d), 1 to 3 y (62.5 μg/d), and 4 to 8 y (75 μg/d) set by the IOM are slightly higher than those set by the SACN (25, 25, and 50 μg/d, respectively) and EFSA (25, 35, and 50 μg/d, respectively) (Table 4).

**TABLE 4:** Recommended intake and ULs for vitamin D

Organization	SACN (2016) [20]		IOM^1^ (2011) [22]		EFSA (2012 and 2018) [101,102]	
	Vitamin D intake (μg/d)					
Age	RNI	UL	RDA	UL	AI	UL
0–6 mo	8.5–10^2^	25	10	25	NE	25
7–11 mo	8.5–10^2^	25	10^3^	37.5^3^	10	35
1–3 y	10	50	15	62.5	15	50
4–10 y	10	50	15	75^5^	15	50
11–17 y	10	100	15	100	15	100
≥18 y^6^	10	100	15^4^	100	15	100

Abbreviations: AI, adequate intake; EFSA, European Food Safety Authority; IOM, Institute of Medicine; NE, not established; RDA, recommended dietary allowance; RNI, reference nutrient intake; SACN, Scientific Advisory Committee on Nutrition; UL, tolerable upper intake level.

1 IOM was renamed the National Academy of Medicine in 2015.

2 Safe intake set by the Committee on Medical Aspects of Food and Nutrition Policy if insufficient reliable data to establish dietary reference values.

3 For ages 6–12 mo.

4 20 μg/d for age ≥71 y.

5 75 μg/d for ages 4–8 y, 100 μg/d for age ≥9 y.

6 Including pregnant and lactating females.

When evaluating the safety limits of vitamin D intake, it is also important to consider current levels of vitamin D in fortified foods and the prevalence of vitamin D supplement use in the United Kingdom. As previously mentioned, vitamin D fortification of fat spreads and breakfast cereals in the United Kingdom is voluntary and does not impose specific restrictions on the concentration of vitamin D used in these products. Nevertheless, the BNF suggests that 1 portion of fortified breakfast cereals (30 g) and fortified fat spreads (10 g) generally contain 1.4 and 0.8 μg of vitamin D, respectively [104]. Among fortified foods, breakfast cereals are the major source of dietary vitamin D from the fortified food category, contributing ∼27% of daily vitamin D intake in children aged 4–10 y and 11% in adults aged 19 to 64 y [25]. Although fat spreads are voluntarily fortified with vitamin D, their contribution to daily vitamin D intake is only around 2% [25], likely due to their relatively low consumption levels. Regarding supplement use, the proportion of adults taking vitamin D supplements was reported to be 28% among adults aged 19 to 64 y, 35% among those aged 65 to 74 y, and 36% among those aged ≥75 y [25]. Although the NDNS 2025 [25] did not report the percentage contribution of vitamin D supplements to daily vitamin D intake, the mean vitamin D intake from all sources, including dietary supplements, of these 3 age groups (19–64, 65–74, and ≥75 y), was 6.9, 8.9, and 9.5 μg/d, respectively. These values were below the RNI of vitamin D (10 μg/d) and far below the UL of 100 μg/d, indicating that the risk of excessive intake from current fortification approaches and supplement use is low. However, ongoing monitoring and modeling simulations of total vitamin D intake from all sources would be necessary if the fortification strategies were to be expanded.

Concerning the relationship between serum 25(OH)D concentration and potential health risk, the IOM reached a conclusion based on the evidence of observational studies that 50 nmol/L would be needed to cover the requirements of a majority of the population and considered a chronic serum 25(OH)D concentration >125 nmol/L having potential adverse effects to health [22]. In comparison, the SACN and EFSA did not set a specific UL for serum 25(OH)D concentration, but the SACN established a threshold of serum 25(OH)D of 25 nmol/L based on the evidence suggesting that an increased risk of poor musculoskeletal health below this concentration [20], whereas a target of near or above 50 nmol/L was suggested by the EFSA [23]. Although the exact serum 25(OH)D threshold for hypercalcemia remains uncertain, a serum 25(OH)D concentration exceeding 375 nmol/L is suggested to be a hallmark of vitamin D overdose and may increase hypercalcemia risk, although such high serum 25(OH)D concentrations are uncommon [105,106]. Systematic vitamin D food fortification generally maintains safe serum 25(OH)D concentrations. However, concerns exist regarding idiopathic hypercalcemia in genetically predisposed infants, despite limited data on this condition’s prevalence [107]. Moreover, mutations in cytochrome P450 family 2 subfamily R member 1 (*CYP2R1*) gene, which is responsible for 25-hydroxylation of vitamin D, are likely to cause vitamin D deficiency, but such gene mutations are uncommon [108].

#### Efficacy of vitamin D fortification of dairy products to improve vitamin D status

Systematic reviews and meta-analyses of RCTs over the past decade have demonstrated the efficacy of vitamin D-fortified dairy products in improving vitamin D status [[109], [110], [111]]. In addition to that, our recent systematic review and meta-analysis [112] summarized evidence from RCTs related to vitamin D-fortified dairy products, including milk/milk powder (*n =* 26), yogurt/yogurt drinks (*n =* 15), and cheese (*n =* 5). The results confirmed that vitamin D-fortified dairy products have the potential to improve vitamin D status, especially vitamin D-fortified milk/milk powder [mean difference (MD): 16.10 nmol/L, 95% CI: 12.68, 19.53 nmol/L, *I*^2^ = 97%] and yogurt/yogurt drinks (MD: 26.58 nmol/L, 95% CI: 20.52, 32.65 nmol/L, *I^2^* = 96%). However, more evidence is needed to confirm the efficacy of vitamin D-fortified cheese (MD: 16.78 nmol/L, 95% CI: −3.61, 37.16 nmol/L, *I^2^* = 99%). However, Wong et al. [112] also reported that the results of leave-one-out analysis of vitamin D-fortified cheese resulted in a significant positive effect on the serum 25(OH)D concentrations (MD = 24.13 nmol/L, 95% CI: 4.69, 43.58 nmol/L, *I*^*2*^ = 90%), indicating the excluded RCT had a significant influence on the meta-analysis results of vitamin D-fortified cheese. The efficacy of vitamin D-fortified milk and yogurt was supported by previous systematic reviews and meta-analyses [109,111], in which Gasparri et al. [111] investigated vitamin D-fortified yogurts (*n =* 9) and reported a significant increase in serum 25(OH)D concentrations after trials (MD = 31.00 nmol/L, 95% CI: 26.1, 35.91 nmol/L, *I*^*2*^ = 100%). Similarly, Cashman and O’Neill [109] examined 4 types of dairy products, including fluid and malted milk [*n =* 13, weighted mean difference (WMD) = 20.3 nmol/L, 95% CI: 13.2, 27.4 nmol/L, *I*^*2*^ = 98%], powdered milk (*n =* 5, WMD = 9.4 nmol/L, 95% CI: 4.0, 14.8 nmol/L, *I*^*2*^ = 81%), cheese (*n =* 3, WMD = 34.9 nmol/L, 95% CI: 8.5, 61.3 nmol/L, *I*^*2*^ = 99%), and yogurt (*n =* 5, WMD = 18.1 nmol/L, 95% CI: 13.6, 22.5 nmol/L, *I*^*2*^ = 56%). However, their results regarding vitamin D-fortified cheese differed from those reported by Wong et al. [112].

It is worth highlighting that the results from systematic reviews and meta-analyses all suffered from high heterogeneity (≥75%) [113], which may be attributed to factors such as the difference in vitamin D dose, intervention duration, and ethnicity, although the heterogeneity may be reduced by subgroup analysis of vitamin D dose and the form of vitamin D [109]. In addition to high heterogeneity being one of the limitations, these systematic reviews and meta-analyses also commented that many RCTs failed to adequately report compliance information, the types of vitamin D and methods for fortification, as well as verify vitamin D doses [109,110,112]. Furthermore, the methods for measuring serum 25(OH)D concentration were also not standardized in the included RCTs [109,110]. Despite limitations, evidence from systematic review and meta-analysis of RCTs has consistently shown that vitamin D-fortified dairy products are efficacious in improving serum 25(OH)D concentration.

#### Real-world example: successful experience in Finland

Countries with vitamin D fortification policies on milk and milk products [[114], [115], [116], [117], [118], [119]] and the characteristics of these policies are summarized in Table 5 [29,54,[114], [115], [116], [117], [118], [119], [120]]. Among those countries, SACN [29] reported that only Finland has evaluated the effectiveness of its fortification policy on population vitamin D intake and status. In 2010, the Finnish National Nutrition Council decided to double the fortification concentration to 1 μg/100 mL for fluid milk products and 20 μg/100 g for fat spreads to ensure sufficient vitamin D intake [121]. The latest update on the fortification policy was in 2016, in which homogenized skimmed milk, including organic skimmed milk, was required to be fortified with ≥1 μg/100 mL of vitamin D_3_ [54,120]. The current recommended total vitamin D intake in Finland is 10 μg/d for individuals 1 to 74 y of age including pregnant and lactating females, and 20 μg/d for those ≥75 y [122].

**TABLE 5:** Summary of current vitamin D fortification policy on dairy products in 8 countries

Country	Fortification policy	Fortification vehicles	Concentration of vit D	Form of vit D
Australia [114]	Mandatory	Edible oil spreads or margarine	≥55 μg/kg	Not specified
Canada [115]	Mandatory	Cow’s milk	2 μg/100 mL	D_2_ or D_3_
		Margarine	26 μg/100 g	
	Voluntary	Goat’s milk	2 μg/100 mL	
Sweden [29,116]	Mandatory	<3% fat milk	0.95–1.10 μg/100 g	Not specified
		<3% fermented dairy products	0.75–1.10 μg/100 g	
		Margarine, fat spreads, and fluid margarine	19.5–21.0 μg/100 g	
Finland [54,120]	Voluntary	Fluid milk products (organic excluded)	1 μg/100 g	D_3_
		Fat spreads	20 μg/100 g	
	Mandatory	Homogenized skimmed milk (organic included)	≥1 μg/100 g	
Norway [29,54]	Voluntary	Butter and margarine	10 μg/100 g	Not specified
		Extra low-fat milk (lactose-free included)	0.4 μg/100 g	
United States [29,117]	Voluntary	Fluid milk	≤2.1 μg/100 g	D_3_
		Yogurt	≤2.23 μg/100 g	
		Margarine	≤8.23 μg/100 g	
Belgium [29,118]	Mandatory	Fat spreads and margarine	6.5–7.5 μg/100 g	Not specified
	Voluntary	Fortified milk	0.75–3.1 μg/100 g	
		Growing-up milk		
		Dairy drinks, desserts		
Chile [29,119]	Mandatory	Liquid milk	≥1 μg/100 mL^1^	D_3_
		Milk powder	≥10 μg/100 g^1^	

Abbreviation: Vit, vitamin.

1 Can be exceeded by ≤40%.

Voluntary vitamin D fortification of dairy products has been successful in increasing vitamin D intake and improving vitamin D status in Finland. Jääskeläinen et al. [32] assessed the effectiveness of the vitamin D fortification on vitamin D status and intake between 2000 (before vitamin D fortification) and 2011 (after fortification) in a representative adult Finnish population (*n =* 3328). After the implementation of the fortification policy, the mean vitamin D intake significantly increased from 7 μg/d for males and females to 14 μg/d for males and 12 μg/d for females. A significant 18 nmol/L increase in serum 25(OH)D concentration from 47.6 to 65 nmol/L was recorded in 2011. The prevalence of insufficient serum 25(OH)D concentration (<50 nmol/L) had significantly dropped from 56% of the population in 2000 to 9% in 2011. Despite the prevalence of vitamin D supplement use increased significantly from 11% in 2000 to 41% in 2011 as a result of the changes in the recommendation for vitamin D supplementation, Jääskeläinen et al. [32] also reported that of people in Finland who did not use vitamin D supplements but consumed milk products, fat spreads and fish based on the Finnish nutrition recommendations, 91% had reached serum 25(OH)D >50 nmol/L in 2011. Additionally, Raulio et al. [123] also investigated the vitamin D intake and vitamin D status in Finnish adults using data from the National Findiet 2012 survey. Similar findings were shown including the mean vitamin D intake of males (17 μg/d) and females (18 μg/d) met the 2010 recommended vitamin D intake (10 μg/d), and the mean of serum 25(OH)D concentration of males (63.3 nmol/L) and females (66.5 nmol/L) reached a sufficient level (>50 nmol/L) [123].

#### Modeling study: simulation of vitamin D fortification of dairy products

The SACN [29] recommended conducting modeling exercises to identify suitable vehicles and determine safe vitamin D concentrations that would effectively improve population intake and status. Although some studies have examined milk using representative United Kingdom population data [124,125], modeling studies specifically investigating dairy products as fortification vehicles remain limited.

Allen et al. [124] conducted a modeling study using milk for simulation, based on representative United Kingdom population data (*n =* 2127) from the first 2 y (2008–2010) of the NDNS Rolling Programme. Table 6 summarizes the food consumption data for estimating vitamin D intake and the definition of food vehicles in the simulation. Results showed that using milk with 1 μg/100 mL of vitamin D reduced the population with intake below RNI from 93% (3.7 ± 3.0 μg/d) to 86% (5.5 ± 3.4 μg/d), and increased mean winter serum 25(OH)D concentration from 39 to 42 nmol/L (Table 7). In addition, milk with 2 μg/100 mL reduced the population with intake below RNI from 93% (3.7 ± 3.0 μg/d) to 73% (7.4 ± 4.4 μg/d), and improved mean winter serum 25(OH)D concentration from 39 to 45 nmol/L (Table 7).

**TABLE 6:** Definitions of food consumption data and foods vehicle in 2 modeling studies

Study	Consumption data for estimating vitamin D intake	Defined food vehicle
Allen et al. [124]	Dietary intake of • Milk and milk-containing foods, • Vitamin D (from fortified foods and supplements) from NDNS (2008–2010)	Milk and milk-containing foods: • Milk as a drink • Milk on cereal • Milk shakes • Hot chocolate (excluding cream, cheese, and yogurt)
Weir et al. [125]	Vitamin D intake and mean milk intake^1^ (portion size (g) per eating occasion) • Whole milk (∼250 g) • Semiskimmed milk (∼180 g) • Skimmed milk (∼160 g) • 1% milk (∼220 g) from NDNS (2008/2009–2011/2012)	Milk: • Whole milk • Semiskimmed milk • Skimmed milk • 1% milk

Abbreviation: NDNS, National Diet and Nutrition Survey.

1 Estimated from graph.

**TABLE 7:** Results of modeling vitamin D fortification of milk from Allen et al. [124] and Weir et al. [125]

Age groups (y, gender)	Vit D intake^1^ (μg/d)		% of meeting RNI (10 μg/d)		Est. serum 25(OH)D concentration (nmol/L)^2^	
Study	[124]	[125]	[124]	[125]	[124]^7^	[125]
No fortification						
1.5–3 (All)^3^	2.5 (2.6)	2.0 (2.1)	7.0	2.0	38 (34–42)	—
4–8 (All)	2.7 (1.9)	—	—	—	38 (34–43)	—
9–49 (Male)	3.1 (2.4)	—	—	—	39 (34–43)	—
9–14 (Female)	2.6 (2.2)	—	—	—	38 (34–42)	—
15–49 (Female)^4^	3.0 (2.6)	2.3 (1.7)	3.0	0.7	39 (34–43)	—
50–64 (All)	5.0 (3.8)	—	10.0	—	41 (36–47)	—
≥65 (All)	5.0 (4.1)	3.4 (2.4)	11.0	1.7	46 (38–56)	—
Overall	3.7 (3.0)	2.5 (1.9)	7.0	0.9	39 (35–45)	—
Simulation: 1 μg/100 mL [124] or 1 μg/100 g [125] of vit D of milk						
1.5–3 (All)^3^	5.6 (3.1)	4.8 (2.6)	23.0	4.2	42 (37–48)	—
4–8 (All)	5.2 (2.6)	—	—	—	42 (36–47)	—
9–49 (Male)	4.9 (3.1)	—	—	—	41 (36–47)	—
9–14 (Female)	4.2 (2.7)	—	—	—	40 (35–46)	—
15–49 (Female)^4^	4.3 (2.9)	3.4 (2.0)	5.0	1.6	40 (36–46)	—
50–64 (All)	6.8 (4.2)	—	20.0	—	44 (38–51)	—
≥65 (All)	7.3 (4.4)	5.3 (2.8)	20.0	6.1	50 (41–61)	—
Overall	5.5 (3.4)	4.2 (2.5)	14.0	3.0	42 (37–49)	—
Simulation: 2 μg/100 mL [124] or 2 μg/100 g [125] of vit D of milk						
1.5–3 (All)^3^	8.7 (4.9)	7.6 (4.4)	56.0^5^	25.7	47 (40–55)	—
4–8 (All)	7.6 (3.9)	—	—	—	46 (39–53)	—
9–49 (Male)	6.7 (4.5)	—	—	—	44 (38–51)	—
9–14 (Female)	5.8 (3.7)	—	—	—	43 (37–49)	—
15–49 (Female)^4^	5.7 (3.6)	3.5 (2.7)	12.0	5.0	42 (37–49)	—
50–64 (All)	8.5 (4.9)	—	32.0	—	47 (40–55)	—
≥65 (All)	9.5 (5.1)	7.1 (3.7)	36.0	17.8	55 (43–67)	—
Overall	7.4 (4.4)	5.9 (3.8)	27.0^6^	12.3	45 (39–53)	—

Abbreviations: 25(OH)D, 25-hydroxyvitamin D; Est, estimated; RNI, reference nutrient intake; Vit D, vitamin D.

1 Presented in mean (SD).

2 Presented in mean (95% confidence interval).

3 1–3 (All) for Weir et al. [125].

4 16–49 (Female) for Weir et al. [125].

5 1% exceeded the tolerable upper intake level of 25 μg/d.

6 0.04% exceeded the tolerable upper intake level of 50 μg/d.

7 Estimated winter serum 25(OH)D concentration.

Similarly, Weir et al. [125] used the NDNS (years 1–4) data to estimate nationally representative vitamin D intake and typical milk consumption of the United Kingdom population (*n =* 4156), in which cow’s milk, including whole, semiskimmed, and skimmed milk, was investigated in 3 vitamin D concentrations (i.e., 1, 1.5, and 2 μg/100 g) (TABLE 6, TABLE 7). Results showed that 1, 1.5, and 2 μg/100 g of vitamin D were theoretically effective in increasing mean vitamin D intake from 2.03 μg/d to 3.69, 4.42, and 5.11 μg/d respectively, without exceeding the UL. However, Weir et al. [125] did not simulate the effect on the population serum 25(OH)D concentration. It is also worth noting that dairy food consumption (g/d) varied across age groups, in which United Kingdom adolescent girls (aged 11–18 y) having the lowest [25]. Therefore, it is rather important to ensure that the concentration of vitamin D fortification in a wide range of dairy products is efficacious in increasing vitamin D intake in this age group. Alternatively, approaches to increase dairy consumption in this age group should be considered. In general, the vitamin D intakes among females aged 9 to 14 and 15 to 49 y were the lowest both at baseline (no fortification) and after fortification simulations (Table 7). Although vitamin D fortification simulations increased vitamin D intake in these age groups, the increase was marginal and the percentage of meeting vitamin D RNI and serum 25(OH)D concentrations was also the lowest in these age groups (Table 7).

For future research, representative United Kingdom population modeling studies investigating vitamin D fortification of a wide range of dairy products are needed to simulate the effectiveness of fortification of different vitamin D concentrations and various types of dairy products on improving vitamin D intake and status. This would provide evidence of the concentration of vitamin D needed for fortification to ensure most of the population can achieve the vitamin D RNI (10 μg/d) and estimate the potential risk of vitamin D overdose. In addition, given there are groups in the United Kingdom population who are more vulnerable to vitamin D deficiency, for example, adolescent girls, darker-skinned individuals and older individuals, modeling studies need to ensure vitamin D fortification can address those at-risk groups to achieve sufficient vitamin D intake and status, especially during winter.

Modeling studies should use data that are representative of the United Kingdom population (e.g., NDNS). Factors including vitamin D intake from natural foods, fortified foods, and supplements, as well as vitamin D status (i.e., serum 25(OH)D concentrations) should also be taken into consideration in the modeling exercises. In addition, the proportion of individuals who do not consume dairy products should be considered when estimating the potential population coverage of vitamin D fortification of dairy products. According to the latest NDNS survey in 2025 [25], ∼3% of the United Kingdom population self-reported as vegetarian and 0.4% as vegan. Although nearly 97% of the United Kingdom population self-reported as nonvegetarian or nonvegan, this does not necessarily mean that all individuals in this group consume dairy products. Therefore, data on the dietary intake of dairy products, including milk, yogurt, and cheese, would be useful for a more realistic estimation of the potential impact of vitamin D fortification of dairy products.

#### Evaluation of the cost-effectiveness of vitamin D fortification of dairy products

Although systematic micronutrient fortification is widely recognized as a cost-effective public health intervention [107,126], few studies have evaluated the cost-effectiveness of vitamin D fortification of dairy products using representative United Kingdom population data. Several studies have been conducted in the EU, for example, Ethgen et al. [127], Sandmann et al. [128], and Hiligsmann and Reginster [129], and Pilz et al. [107] highlighted that most of these studies assessed the cost-effectiveness of vitamin D fortification in the elderly population rather than the whole population. In the United Kingdom, further research is needed to identify the true burden of childhood rickets and osteomalacia in adults and compare the cost-effectiveness of fortification/supplementation approaches [130], which may be based on the relationship between serum 25(OH)D concentration and the risk of rickets and osteomalacia.

Concerning the economic aspects of vitamin D deficiency in the United Kingdom, few studies conducted in the United Kingdom to evaluate the economic burden of vitamin D deficiency and the economic benefits of improving vitamin D status. Kamudoni et al. [131] evaluated the NHS health expenditures of vitamin D deficiency in pregnant females in England and Wales. As part of the evaluation, they conducted a meta-analysis of health complications associated with vitamin D deficiency in pregnant females, including preeclampsia, preterm birth, and small for gestational age (SGA) using RCTs and observational studies. Results of meta-analysis of RCTs showed that vitamin D supplementation could reduce the risk of preeclampsia significantly [*n =* 5, odds ratio (OR) = 0.75, 95% CI: 0.662, 0.843, *I*^*2*^ = 0%] whereas there was no association found with preterm birth (*n =* 4, OR = 0.783, 95% CI: 0.49, 1.251, *I*^*2*^ = 33%) and SGA (*n =* 4, OR = 0.76, 95% CI: 0.38, 1.25, *I*^*2*^ = 12%). It was estimated that the treatment cost for preeclampsia was £9278 per case and the total cost to the NHS in England and Wales was £272.68 million. Kamudoni et al. [131] also estimated that about 4130 cases of preeclampsia could be avoided by addressing pregnant females with suboptimal vitamin D status and would also result in a net saving of £18.6 million for the NHS in England and Wales. Another United Kingdom–based study by Poole et al. [132] evaluated the cost-effectiveness and budget of empirical vitamin D supplementation (20 μg/d) treatment to prevent unintentional falls in older adults (≥60 y). Results showed that those with vitamin D supplementation over 5 y could significantly prevent major (190,000 cases) and minor falls (430,000 cases), acute deaths (1579 cases), 840,000 person-years of long-term care, and 8300 deaths associated with increased mortality in long-term care. Besides, a substantial cost could also be saved by fall prevention with the estimation of a net saving of £420 million. Similarly, Lacey et al. [133] evaluated the cost-effectiveness of vitamin D supplementation for reducing the risk of all-cause mortality, hip fractures, and nonhip fractures in Irish adults with vitamin D deficiency [serum 25(OH)D <30 nmol/L] across 3 age groups, including ≥50, ≥60, and ≥70 y. The study found that the cost/quality-adjusted life years (QALY) in all 3 groups were below the acceptable cost-effectiveness threshold of €20,000/QALY, indicating vitamin D supplementation was cost-effective, and it was the most cost-effective in adults ≥70 y at €5400/QALY. Furthermore, Aguiar et al. [134] simulated different population strategies to prevent vitamin D deficiency in England and Wales (*n =* 58,381,200) over 90 y, including wheat flour fortification (10 μg/100 g), free vitamin D supplementation to at-risk groups, a combination of both strategies, and a no-intervention scenario. Results showed that wheat flour fortification was the most cost-effective as the cost savings from preventing vitamin D deficiency were more than the implementation costs of fortification, with an estimated reduction of 25% cases of vitamin D deficiency. Furthermore, Aguiar et al. [134] suggested that the vehicle for fortification needs to be largely consumed by the target population and the price of the fortified food products should be kept low as there may be a food access barrier for those who have a lower income. Notably, none of the studies examined the economic burden of rickets and osteomalacia, given an increasing trend in the hospitalization rate for rickets in children [27,28].

In addition to evaluating the cost-effectiveness of vitamin D fortification of dairy products, Buttriss et al. [135] highlighted the need to investigate the “real-world” economic implications of vitamin D fortification for industry and stronger collaboration among academics, policymakers, and industry to make progress. Several factors affect the cost of food fortification for manufacturers, including the fortificant type, the processing stage at which it is added, quality control requirements, order volumes, specific food category, and the profit margin between and within different food categories [135]. The technology needed for fortification would contribute to the cost; for example, vitamin D is one of the most expensive fortificants added to breakfast cereals, not due to the price of vitamin D itself but because of the expensive technologies needed for its addition [135]. It is however believed that the cost of fortifying dairy products with vitamin D is less expensive and not likely to be a huge economic burden [135]. An economic modeling study by Vičič et al. [136] focusing on Slovenian females aged 44 to 65 y estimated that the annual cost of achieving 10 μg of vitamin D/d/person through direct fortification of milk and yogurt was €0.09, indicating that it was a cost-effective approach. The fortification cost was calculated based on the vitamin D prices provided by suppliers of nutritional products.

Overall, the evidence suggests that systematic approaches to improve vitamin D status are likely to be cost-effective to help reduce the prevalence of vitamin D deficiency, as well as saving £ millions of expenses on vitamin D deficiency-related outcomes, although there is only 1 study [134] that investigated a food fortification approach. However, further research is required, given there is a lack of evidence regarding vitamin D fortification of dairy products in the United Kingdom, including a modeling study of the cost-effectiveness of this approach, the economic burden of rickets and osteomalacia, as well as other vitamin D deficiency-related health implications, and estimation of the cost of fortification for the dairy industry.

### Future directions

Although there is strong evidence that suggests the improvement in vitamin D status in the United Kingdom population is essential, there are research gaps identified that may need to be addressed in relation to the legislation and promotion of vitamin D fortification of dairy products. First, as recommended by the SACN [29], conducting modeling studies on vitamin D fortification of a wide range of dairy products using representative United Kingdom population data (e.g., NDNS) is necessary. This evidence helps identify the appropriate, effective, and safe vitamin D concentrations for dairy products fortification, which would help the United Kingdom population, especially those who are at risk of vitamin D deficiency, to attain the vitamin D RNI of 10 μg. Second, it is important to assess the financial burden associated with suboptimal vitamin D intake and status and especially evaluate the cost-effectiveness of vitamin D fortification of dairy products. Third, investigating the consumer preferences and WTP toward vitamin D fortification and vitamin D-fortified dairy products in the United Kingdom, which may help identify the factors that hinder the acceptance of vitamin D-fortified dairy products. Therefore, appropriate strategies to address consumers’ concerns can be implemented. Fourth, analyzing the potential cost for dairy processors associated with vitamin D fortification, for example, the equipment needed for vitamin D fortification, may give information on the impact on the industry. Last but not least, identifying factors that influence the attitudes of retailers toward and their willingness to stock and promote vitamin D-fortified dairy products. Addressing those factors may increase consumers’ exposure to vitamin D-fortified dairy products and, consequently, the likelihood of purchase.

In conclusions, clear evidence indicates a high prevalence of inadequate vitamin D intake and suboptimal vitamin D status across all age groups in the United Kingdom. Additionally, with the ongoing decline in red meat consumption and the increasing shift toward plant-based diets may further reduce vitamin D intake and status within the United Kingdom population. Therefore, strategies are needed to prevent further declines in vitamin D status. The SACN has now brought systematic vitamin D fortification in the United Kingdom to attention. One possible approach is the systematic fortification of dairy products with vitamin D, as dairy remains a staple food in the United Kingdom, and it has been a successful strategy in other countries. However, several challenges exist in putting this strategy into practice. This paper reviewed current evidence on 5 potential barriers to promoting and introducing mandatory vitamin D fortification of dairy products in the United Kingdom. Robust and sufficient evidence must be provided to policymakers to demonstrate both the economic and practical benefits of vitamin D fortification in dairy products. However, given the potential lengthy legislation process and uncertainties surrounding fortification policy direction and progression, individuals currently at risk of vitamin D deficiency and with low intake of vitamin D remain vulnerable unless interim strategies are implemented. Educational campaigns would be necessary to raise awareness and knowledge of the benefits and functions of vitamin D. These campaigns may help alleviate concerns about vitamin D fortification and fortified foods and potentially increase public acceptance. In addition, dairy food manufacturers and retailers would play an important role in supplying and promoting vitamin D-fortified dairy products. However, the potential costs to dairy food processors are uncertain as vitamin D fortification may require specific equipment. Ultimately, consumer demand may be one of the key factors that influence the willingness of retailers to stock and promote vitamin D-fortified dairy products, but further investigation is needed to confirm and identify other possible factors.

## Author contributions

The authors’ responsibilities were as follows – CLW, DIG, DA, AMT, JG: contributed to the conception and design of the manuscript; CLW, DIG: prepared tables and figures; CLW: was under the supervision of JG; and all authors: contributed to the writing, editing and reviewing of the manuscript, read and approved the final manuscript.

## Data availability

Data described in the manuscript will be made available upon request pending.

## Funding

This work was supported by the Consumer Lab funded by the Biotechnology and Biological Sciences Research Council (BBSRC), with support from the Department for Environment, Food and Rural Affairs (DEFRA), Innovate United Kingdom and the Medical Research Council (MRC) (grant reference BB/X010805/1).

## Declaration of Generative AI and AI-Assisted Technologies in the Writing Process

The author(s) declare that no generative AI or AI-assisted technologies were used in the writing of this manuscript.

## Conflict of interest

The authors declare that this research was supported by a grant from the Consumer Lab funded by the Biotechnology and Biological Sciences Research Council, which provided financial support for workshops and the employment of a research assistant (C.L.W.). The funding body had no role in the study design, data collection and analysis, decision to publish, or preparation of the manuscript. The authors also declare that A.M.T., from Valio Ltd. (Finland), was our formal partner, providing industry input and in-kind analytical support. Author D.I.G. also declares that he has received travel expenses and honoraria in connection with lectures and meetings from the UK Dairy Council (now Dairy UK), the Dutch Dairy Association, the European Dairy Association, the International Dairy Federation, Centre National Interprofessionnel de l’Industrie Laitière, and the US Dairy Research Institute. Author L.K.P. has previously received research funding and honoraria (speaker expenses) from the Dairy Council for Northern Ireland.
